# Functional Imaging Reveals Numerous Fields in the Monkey Auditory Cortex

**DOI:** 10.1371/journal.pbio.0040215

**Published:** 2006-06-20

**Authors:** Christopher I Petkov, Christoph Kayser, Mark Augath, Nikos K Logothetis

**Affiliations:** **1**Max Planck Institute for Biological Cybernetics, Tübingen, Germany; McGill UniversityCanada

## Abstract

Anatomical studies propose that the primate auditory cortex contains more fields than have actually been functionally confirmed or described. Spatially resolved functional magnetic resonance imaging (fMRI) with carefully designed acoustical stimulation could be ideally suited to extend our understanding of the processing within these fields. However, after numerous experiments in humans, many auditory fields remain poorly characterized. Imaging the macaque monkey is of particular interest as these species have a richer set of anatomical and neurophysiological data to clarify the source of the imaged activity. We functionally mapped the auditory cortex of behaving and of anesthetized macaque monkeys with high resolution fMRI. By optimizing our imaging and stimulation procedures, we obtained robust activity throughout auditory cortex using tonal and band-passed noise sounds. Then, by varying the frequency content of the sounds, spatially specific activity patterns were observed over this region. As a result, the activity patterns could be assigned to many auditory cortical fields, including those whose functional properties were previously undescribed. The results provide an extensive functional tessellation of the macaque auditory cortex and suggest that 11 fields contain neurons tuned for the frequency of sounds. This study provides functional support for a model where three fields in primary auditory cortex are surrounded by eight neighboring “belt” fields in non-primary auditory cortex. The findings can now guide neurophysiological recordings in the monkey to expand our understanding of the processing within these fields. Additionally, this work will improve fMRI investigations of the human auditory cortex.

## Abstract 2

High-resolution fMRI reveals a detailed functional description of auditory cortical fields in the macaque monkey.

## Introduction

The primate brain consists of numerous fields with specific roles in sensing and perceiving the environment. Concerning the cortical regions involved in the perception of sound, anatomical studies suggest that there are around a dozen auditory cortical fields (ACFs) [ 1– 5]. Based on their subcortical input, these ACFs embody the initial stages of sound processing at the cortical level. However, for many of the fields either their role in acoustical processing comes from limited functional evidence in select primate species or is simply unknown. In the latter case, the anatomical evidence for the existence of a particular functional field remains unconfirmed. This not only leaves us with an incomplete understanding of auditory cortex, but also impedes our understanding of subsequent processing relying on input from these ACFs, such as by fields specialized for processing communication sounds or those involved in integrating information from the different senses.

Functionally locating the many primate ACFs is a necessary first step towards understanding their role in audition. In this regard, functional magnetic resonance imaging (fMRI) is promising because its extensive field of view and high spatial resolution can map the function of numerous fields. For example, visual scientists use fMRI to reliably segregate many visual fields in both human and monkey subjects, a process that is at the core of visual functional imaging studies (for reviews see [ 6, 7]). Unfortunately, human auditory fMRI has struggled for over a decade to locate many of the auditory fields [ 8– 15], and auditory fMRI of other primates was unavailable for guidance or comparison. A recent human functional imaging study provided compelling evidence for two human ACFs [ 13]. There the authors used high-resolution fMRI (i.e., high-magnetic field imaging at 7-Tesla [T]) to functionally describe two auditory “core” or primary cortical fields, the possible human homologues of primate fields A1 and R. The authors also defined a common border between these fields. Often, however, it is not possible to identify specific fields with the reported activity [ 8– 12, 14, 15]. Thus many human ACFs remain unidentified and cannot be linked to specific auditory operations.

The small scale and close proximity of the many expected functional ACFs could be one explanation for the difficulty in localizing them. To overcome this problem, high-resolution imaging of humans at high-magnetic fields will certainly help [ 13]. However, other problems cannot be solved solely with improved imaging technology. Specifically, the functional properties of certain ACFs are mainly known from neurophysiological recordings in nonhuman primates like the macaque monkey [ 16– 21], and homologies between humans and other primates are only beginning to be established [ 13, 22, 23]. Thus it is difficult to understand how to rely on monkey neurophysiology to clarify or guide human functional imaging. We reasoned that imaging the macaque auditory cortex would be invaluable for functionally localizing ACFs. First, the more extensive anatomical and neurophysiological data in these species [ 1, 3, 16– 21] can identify the source of localized fMRI activity. Then, upon an understanding of how fMRI results reflect already described properties, the imaging method has the potential to reveal and describe ACFs with currently unknown functional properties.

We used high-resolution fMR at 4.7- and 7-T to image the auditory cortex of behaving and of anesthetized macaque monkeys. To functionally localize and describe many auditory fields we relied on prominent organizational properties, including suggested processing differences between primary and non-primary auditory cortical fields [ 1, 16– 21]. In particular, one general property that we relied on is neurons' selectivity or tuning for sound frequency. The progression of neuronal frequency selectivity across an auditory field is known as tonotopy, as will be called here, or cochleotopy, a topographical representation of the cochlea which is analogous to retinotopy in the visual system [ 6, 24]. Neurophysiological studies commonly use tonotopic gradients based on neuronal frequency tuning to identify the better studied fields in auditory cortex such as A1 [ 16– 18, 20, 21, 25]. Similarly, fMRI activity from different frequency selective regions along a field's tonotopic gradient can be used to identify that particular ACF. However, limited fMRI maps of frequency selectivity, such as those arising from one or a few ACFs, can be ambiguous [ 12]. This is because the frequency selectivity of neurons in a neighboring field could elicit a similar activity pattern.

To associate activation patterns with multiple ACFs at once, experiments were designed to activate large portions of auditory cortex while preserving neuronal frequency tuning of the active ACFs. This was achieved in part by optimizing stimulation procedures specifically for fMRI, even if (1) such stimulation might not have a priori been expected to elicit strong responses from many neurons in auditory cortex, or (2) the intensity levels used (70–85 dB sound pressure level [SPL]) would have been expected to degrade neuronal frequency tuning. As a result, we were able to activate large portions of auditory cortex and to functionally tessellate many auditory cortical fields in anesthetized and in behaving monkeys.

## Results

Our initial goal was to activate a large region of the macaque auditory cortex upon which many ACFs could be functionally localized and described. For initial experiments we used sounds containing a broad frequency spectrum (broadband noise, 0.250–19 kHz) to stimulate many auditory neurons. Then, with carefully selected sound intensities, we used low- and high-frequency sounds to reveal multiple frequency selective regions in auditory cortex. The activity patterns observed then served as a basis for more detailed studies using up to six different sound frequencies. Although single-frequency tones are not considered a strong stimulus for non-primary auditory cortex neurons, moderate to high-intensity tones (70–85 dB SPL) elicited robust and spatially specific fMRI blood-oxygen level dependent (BOLD) responses throughout auditory cortex. Thus, tonal stimulation allowed us to functionally tessellate large portions of auditory cortex. Broader bandwidth sounds (band-passed noise, see “Sound stimuli and preparation” in Materials and Methods) varying in frequency content were also used and showed similar functional tessellation results as tones.

### Optimizing Auditory Cortex Activity

**Figure 1:**
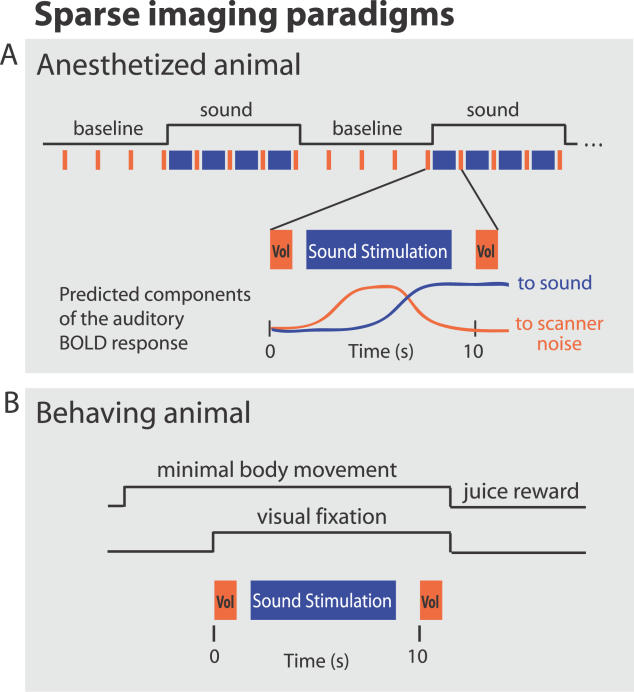
Sparse-Imaging Paradigms (A) The sparse-imaging paradigm for imaging the anesthetized animals (at 4.7- and 7-T) consisted of a block design with alternating stimulation and baseline periods. Data acquisition and sound stimulus were interleaved as schematized in the blow-out of a single sequence during a stimulation block. Here, the sequence initiates with an imaged brain volume, allowing sounds to be presented during the subsequent silent period. Because of the delay in the hemodynamic (BOLD) response the next volume acquired 10 s later reflects the sound stimulation that occurred approximately 6 s before. This minimizes influences on the auditory cortex BOLD response by the scanner noise that occurred 10 s before. This sequence is repeated four times during a stimulation block and four times during a baseline (no-stimulation) block. (B) For imaging the behaving animal (at 7-T), the animal completed behavioral trials composed of the single sparse-imaging sequence during minimal eye and body movements (see “Behaving Animal Preparation and Imaging” in Materials and Methods).

**Figure 2:**
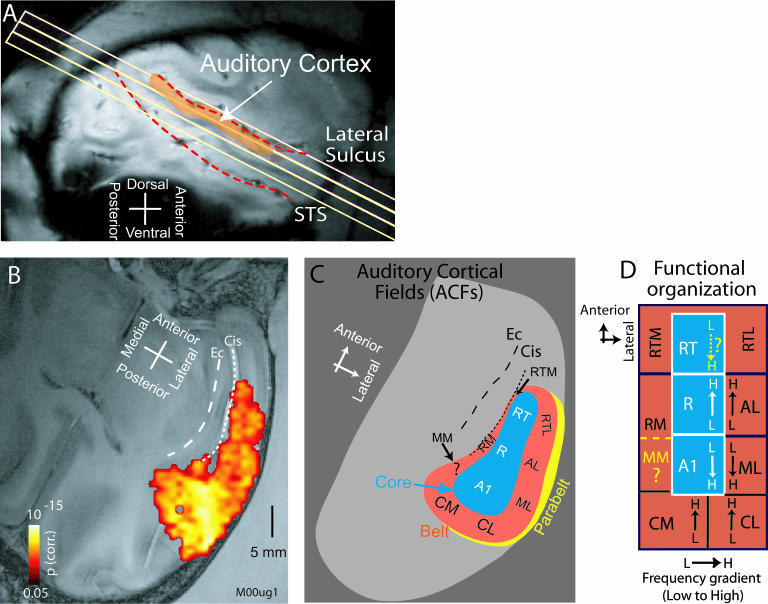
Macaque fMR Imaging and ACFs (A) Imaging slices were aligned with the lateral sulcus to obtain a plane of auditory cortex activity on the lower bank of this sulcus (colored in translucent orange; STS, superior temporal sulcus). (B) Shows activations to broadband noise from one anesthetized animal. (C) Schematizes the possible ACFs contributing to activity in (B) [ 1, 3, 16– 21]. (D) Schematizes functional properties of ACFs that could distinguish them. Two of the three core or primary ACFs (blue) have frequency selective gradients, shown with arrows from low (L) to high (H) frequency selectivity. The question mark in the core field RT indicates limited evidence of such a gradient in primates [ 2]. Anatomy suggests there could be seven or eight belt or non-primary ACFs (orange) [ 1, 3]. Only four of the belt ACFs have neurophysiologically described frequency gradients, and these fields are considered to be less responsive to single-frequency tones, preferring instead more complex sounds [ 16, 18, 19, 21, 28, 29]. Abbreviations: Ec, external capsule; Cis, circular sulcus.

To obtain robust auditory cortex activity despite the noise generated by MR acquisition, we used a sparse-imaging paradigm, which is also known as a “sparse-sampling” paradigm. This paradigm minimizes the influence of the scanner noise on the auditory cortex response and allows sounds to be presented during silent periods between image acquisition [ 10, 13] ( Figure 1). Acoustical stimulation in combination with the sparse-imaging technique was effective in revealing large portions of the monkey auditory cortex ( Figure 2).

The imaging slices were aligned with the lateral sulcus ( Figure 2A). This allowed us to obtain a broad strip of activity onto a slice from auditory cortex, on the lower bank of the lateral sulcus. The activity obtained from an anesthetized animal experiment is shown in Figure 2B. The significantly active voxels covered a large extent of auditory cortex (in Figure 2B, ˜3 cm antero-posteriorly and ˜1.5 cm, full-width, medio-laterally). This activated region reflects the sound response of numerous ACFs ( Figure 2C). Specifically, the auditory “core” or primary auditory cortex is thought to be composed of 3 centrally located ACFs: A1, R, and RT ( Figure 2D, blue) representing the first stage of parallel processing in auditory cortex [ 1– 3, 17– 19, 21]. Of these three fields, the functional properties of field RT remain poorly characterized (see Figure 2D). Anatomical studies also suggest that seven or eight “belt” or non-primary ACFs ( Figure 2D, orange) encircle the core, representing the next hierarchical stage of auditory processing [ 3, 26, 27]. Only four of the belt fields have neurophysiologically described functional properties (fields CM, CL, ML, and AL, see Figure 2D) and the data suggest that neurons in these regions of auditory cortex prefer complex sounds with broader frequency content, being difficult to drive with single frequency tones [ 16, 18, 19, 21, 28, 29].

Having obtained fMRI activity throughout auditory cortex we next aimed to obtain spatially resolved activity patterns from this large region to functionally delineate many ACFs. Sound intensity levels for these experiments were preselected within the range of 70 and 85 dB SPL to obtain broad auditory cortex activity while preserving neuronal tuning for sound frequency (see Materials and Methods).

### Functional Tessellation of Auditory Cortex

**Figure 3:**
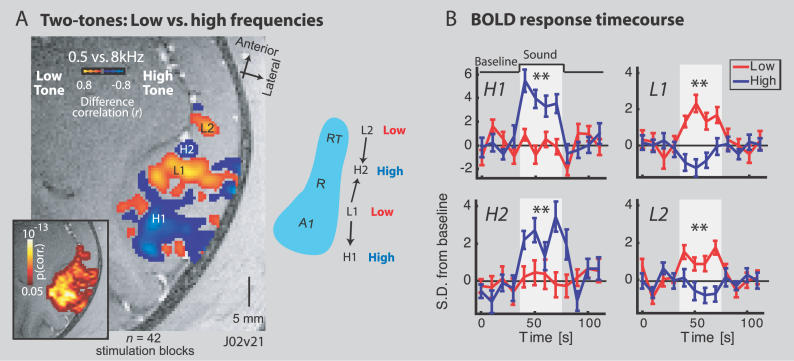
Two Tones Reveal Multiple Frequency Selective Regions over the Auditory Core (A) An experiment with alternating blocks of 0.5 kHz (Low) and 8 kHz (High) frequency tones. First, significantly active voxels to these sounds are identified (see inset). Within this region, individual voxels' frequency selectivity is determined by the difference in the signal correlation to the Low (red to yellow) vs. High (blue to cyan) tones. The results are thresholded to not display weakly frequency selective voxels. Four frequency selective regions are observed: (H1) labels a posterior high-frequency selective region, (L1) a more anterior low-frequency region that could be shared by the anterior portion of A1 and the posterior of R (see schematic of the auditory core to right), (H2) an anterior high-frequency selective region, shared by R and RT, and (L2) the most anterior low-frequency selective region observed, belonging to field RT (also see Figure 2D). (B) Timecourse of the BOLD response illustrating that clusters of voxels underneath the labels in (A) showed greater activation to the high frequency tone at H1 and H2 but greater activation to the low frequency tone at L1 and L2 (see ** during sound stimulation periods; *t*-tests, all *p* < 0.002). Error-bars signify standard error of the mean across stimulation block repeats.

To unambiguously associate activity patterns from the numerous ACFs we aimed to reveal multiple reversals of tonotopic gradients. For example, field A1′s tonotopic gradient predicts a posterior high-frequency selective region and a more anterior low-frequency selective region [ 16– 18, 20, 21]. Field R has a tonotopic gradient that is reversed or mirrored in relation to A1 ( Figure 2D, [ 17, 20]). Additionally, the gradient of field RT, if it exists [ 2], should mirror the gradient in field R. Thus, in the antero-posterior direction, one could expect fMRI activity over these three fields to elicit the following spatially alternating pattern of frequency selectivity, from posterior to anterior: high, low, high, low. Consistent with the prediction, we observed this precise pattern of frequency selectivity ( Figure 3).

The fMRI activation patterns to low and high frequency tones revealed two pairs of high- and low-frequency selective regions as one proceeds from posterior to anterior across auditory cortex ( Figure 3A, labeled as H1, L1, H2, and L2). This pattern of tone activity is consistent with the presence of the predicted frequency selective regions across the three core ACFs: A1, R, and RT. Also, the distance between H1 and L2 in Figure 3A (˜2 cm) corresponds to the scale of the core reported from macaque anatomical studies (1.6–2 cm), with A1 typically being the larger of the three fields in width (˜0.8 cm) and length (˜1 cm, see [ 3]). Therefore, the tone frequency-selective regions, their scale and location all strongly support that these responses emanate from the three core ACFs.

**Figure 4:**
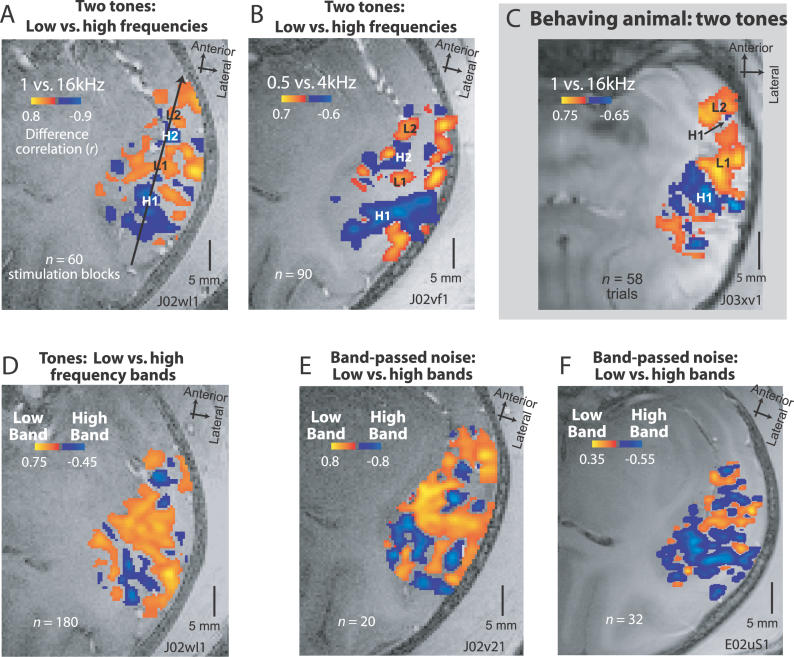
Reliable Frequency-Selective Regions in the Behaving and in the Anesthetized Animals Observed throughout these examples are the two pairs of high and low frequency selective regions as shown in Figure 3, i.e., H1, L1, H2, and L2. (A–C) the results from using two tones. (A–B) show different scanning days with the same anesthetized animal as in Figure 5 (J02, the animal and the experiment are identified in the lower right of each panel). Arrow in (A) shows the axis along which the frequency selective regions lie. (C) Behaving animal results (animal J03). (D–F) Further results with anesthetized animals (J02 and E02). (D) Results using two combinations of tone frequencies (Low: 0.5, 1, and 2 kH tones; High: 4, 8, and 16 kHz tones). (E–F) In two animals these panels reveal the patterns of activity for low and high frequency one-octave band-passed noise (Low: 0.5–1 kHz; High: 4–8 kHz).

The selectivity of these regions to sounds of the specified frequency was statistically significant ( Figure 3B). This alternating pattern of low and high frequency selective regions was also consistently observed across many experiments ( Figure 4): including with the same animal ( Figure 4A and 4B), with a behaving animal ( Figure 4C) and in experiments using different sounds such as combinations of low and high frequency tones ( Figure 4D) or band-passed noise ( Figures 4E and 4F; for a description of all experiments see Protocol S1). Furthermore, in some of these two-tone experiments a low-frequency selective region posterior to H1 was evident, which was the first indication of an extended pattern of frequency selectivity that extended beyond the auditory core (e.g., Figure 4C from the behaving animal). The extended pattern of frequency selectivity was pursued with further experiments.

**Figure 5:**
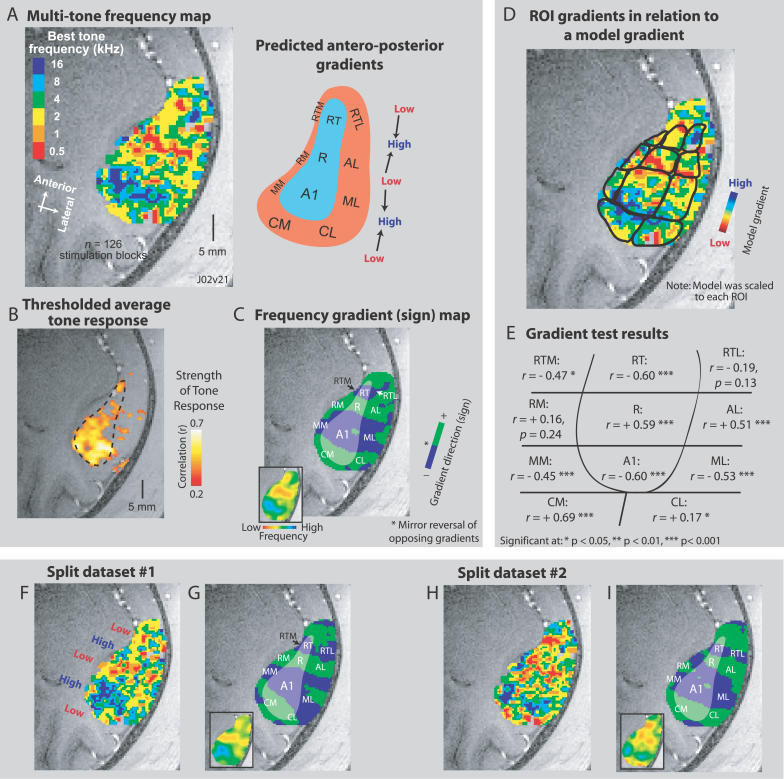
Delineating Borders and ROIs over Auditory Cortex (A) Multiple tone-frequency map, resulting from the presentation of six tone frequencies individually in pseudo-randomized stimulation blocks. The best-tone frequency response of the significantly active voxels is color-coded (see “MRI data analysis” in Materials and Methods). Tone activations were extensive over auditory cortex, see text. Right of (A) schematizes the ACFs that could be active in this broad region including the directions (from Low to High) of the expected frequency selective gradients. (B) The core preferentially responds to tones in comparison to belt regions [ 19, 21]. An “auditory core” ROI was drawn around the average tone response, thresholded to the approximate length (˜1.8 cm) and full width (˜0.8 cm) of the core [ 3]. (C) The results of a gradient analysis that assigns a sign to gradients progressing in the anterior direction, along an axis predetermined by points underneath labels H1 and L2 from the data in Figure 3A. Along this axis, gradients that progress anteriorly from low to high frequencies, ascending gradients, are assigned a positive sign (green) and those from high to low, descending gradients, a negative sign (blue). Multiple mirror reversals of frequency gradients are observed and borders between ACFs are delineated where signs reverse, or the two colors meet. The smoothed version of the multi-frequency map (A) used for the gradient analysis is inset in (C). The area of the “auditory core” ROI from (B) is overlaid in transparent white over the map in (C). (D) Auditory cortex ROIs were delineated relying on the gradient sign map borders, shown here overlaid on the data from the multi-tone frequency map. Each ROI's gradient from the multi-tone data was tested in relation to a model gradient progressing from low to high values anteriorly, see colorbar. (E) Reports the results of the ROI gradient tests as correlation coefficients, *r*, and associated *p*-values. The combined analyses localize many auditory fields with mirror reversed frequency gradients and functionally tessellate large portions of auditory cortex. (F–I) Split datasets from the entire experiment. Format as for panels (A and C).

We next studied responses to multiple tone frequencies (six tone frequencies presented serially, see “Sound stimuli and presentation” in Materials and Methods). The expected frequency selectivity across the core predictably revealed finer patterns of activity ( Figure 5). Importantly, these results helped to localize other fields outside of the core because the responses to tones at these moderate to high sound intensity levels also extended into the auditory belt, where neurons respond to but are largely difficult to drive with single-frequency tones [ 16, 18, 19, 21, 29]. For instance, the most posterior sections of the multi-tone frequency map in Figure 5A (showing the best-frequency response of individual voxels) reveal a descent from high to low frequency selectivity. This is consistent with the known frequency gradients of the posterior belt fields CM and CL. The gradients in these fields mirror the one in A1 and would share with it part of the posterior high-frequency selective region. Additionally, the prominent frequency selective regions extended medio-laterally, further than the width of the core [ 3]. These bands of frequency selectivity resemble neurophysiologically determined iso-frequency bands [ 25, 30]. Their extent laterally is consistent with the frequency gradients that have been reported neurophysiologically for the lateral belt fields CL, ML, and AL [ 18, 19, 21], which are known to be collinear in relation to their medial neighbors CM, A1, and R (see Figure 2D). Interestingly, a medial extent of “iso-frequency” bands was also observed, extending toward medial belt fields with previously unknown functional properties ( Figure 5A, also see Figure 2D).

From the multi-tone data, we next delineated antero-posterior borders between neighboring fields using a gradient analysis method developed to demarcate retinotopic visual field borders [ 24]. Using the gradient analysis, borders can be delineated from mirror-symmetric tonotopic gradients at the point of gradient reversal [ 13], see Figure 5. The voxel-based analysis in Figure 5C assigns a sign, positive or negative, to the two possible directions of frequency gradients in the multi-tone data ( Figure 5A). The antero-posterior axis, along which this gradient analysis was evaluated, was predetermined from points at labels H1 and L2 in the separate dataset ( Figure 3A). The results revealed borders running medio-laterally across auditory cortex ( Figure 5C). These borders run between multiple core and belt regions, subdividing the auditory cortex in the antero-posterior direction. Next, to localize the auditory core in relation to the belt we exploited the finding that the core responds better to tones than the belt [ 11, 19, 21], although with fMRI both regions could respond significantly to tones (see Figure 5A which is based on significantly active voxels). Hence one strategy is to use the strength of the obtained tone response to localize the core. Figure 5B displays a tone activation map where the threshold for activation was chosen so that activations covered an area consistent with the approximate average length (˜1.8 cm) and full width (˜0.8 cm) of the core in these species [ 3]. A region of interest (ROI) was then drawn around the extent of this thresholded activity ( Figure 5B). This “auditory core” ROI was then superimposed on its exact location on the gradient sign map ( Figure 5C), providing a simple method from the available data for approximating the position of the core in relation to the belt (for a different strategy see “Hierarchical Processing in Auditory Cortex” in Results).

### Significance of ACF Gradients

The gradient analyses shown in Figure 5C reveal multiple mirror reversals of frequency gradients in the antero-posterior direction and that adjacent medial, central and lateral regions have collinear frequency gradients. These results suggest that the three centrally located primary ACFs (A1, R, and RT) are surrounded by four lateral (CL, ML, AL, and RTL) and four medial (CM, MM, RM, and RTM) belt fields. This model supports the interpretation of the anatomical data, including the available neurophysiology, that 11 ACFs reside in this region of auditory cortex [ 1]. To test the significance of the frequency gradient within these fields, we used the results from the gradient sign map to outline 11 ROIs corresponding to these presumed fields. These ROIs are shown overlaid on the multi-tone map in Figure 5D. The correlation between the frequency gradient inside each ROI and a model (scaled to the size of each ROI and progressing from low to high values along the posterior to anterior axis) was tested for significance.

The results of the gradient tests for each ROI are shown in Figure 5E and support the presence of the 11 auditory fields. We observe that all of the gradients are in the predicted direction (note the alternating correlation signs from posterior to anterior) and that nine of the 11 ROIs' gradients are significantly related to the model gradient. The remaining two ROIs (representing belt fields RTL and RM) showed some evidence for a gradient in the predicted direction ( Figure 5D). However, these may not have reached significance ( *p*-values of 0.13 and 0.24 respectively) possibly because of their small size and/or suboptimal ROI placement (see “MRI data analysis” in Materials and Methods).

**Figure 6:**
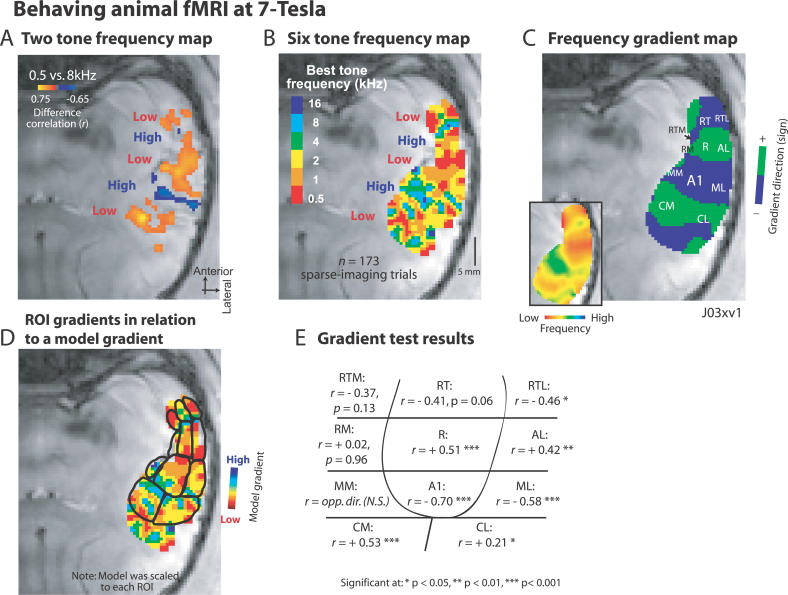
Functional Tessellation of the Behaving Animal's Auditory Cortex (A) Contrasting responses to two tones reveals an extended alternating pattern of (Low, High, Low, High, Low) frequency selectivity. (B) Multi-tone frequency map. (C) Gradient analysis. (D) Delineated auditory cortex ROIs. (E) Statistical results of the ROI gradient tests; format as for Figure 5 (A), (C–E). Note that the *(n)* for these data reflect the number of correctly completed trials, whereas for the anesthetized animal imaging one stimulation block contains four sparse-imaging sequences, or such “trials.”

**Figure 7:**
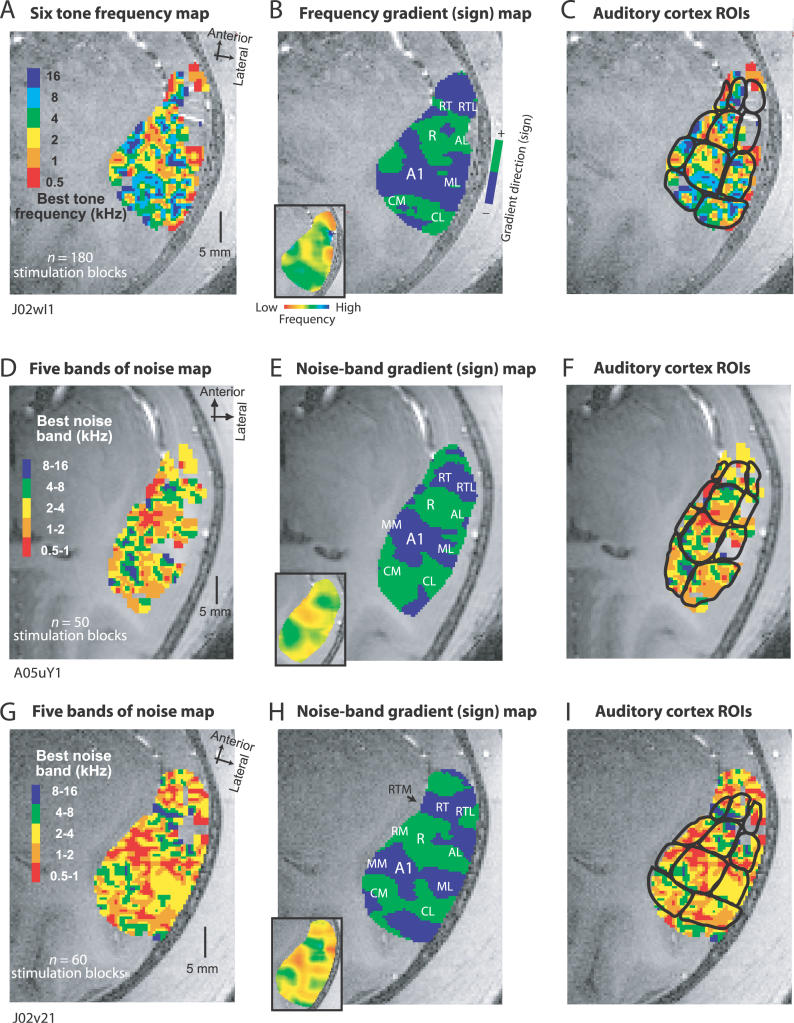
Delineating ACF Borders with Multiple Tones and Bands of Noise (A–C) An additional multi-tone experiment with animal J02. Format as for Figure 5 (A), (C and D). (D–I) Show the results from two animals where five one-octave bands of noise (band-passed noise) were used to localize ACFs. (D and G) Five best noise-band maps showing the antero-posterior frequency gradients. (E and H) show the gradient sign procedure revealing borders between neighboring ACFs. (C, F, and I) show auditory cortex ROI outlines. The results of the gradient tests within these outlines in comparison to the model gradient are shown in Table 1.

Additional evidence supporting the observed ACF patterns was obtained in other experiments. Prominently, with the behaving animal, similar multi-tone and gradient map patterns were observed ( Figure 6). For this animal, the ROI gradient tests revealed that seven of the ROIs had significant gradients, two may have been close to significance (those representing fields RT and RTM) and all, but one (of field MM), were in the predicted direction (see Figure 6D and 6E). Additionally, Figure 7 shows another multi-tone experiment with the anesthetized animal from Figure 5, revealing the reliability of the observed ACF patterns across experiments with the same subject (see Figure 7A–C, also see next section).

**Table 1:**
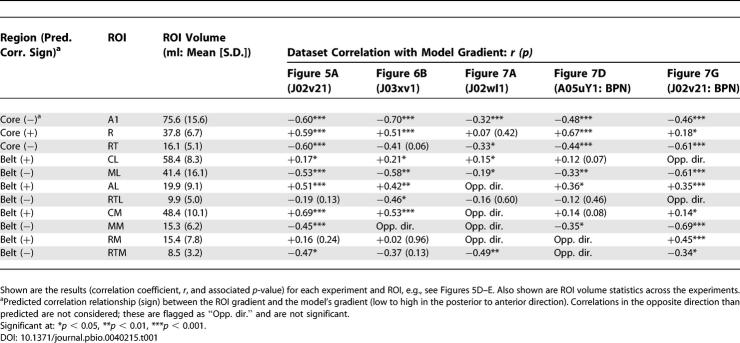
Associations between the Gradients of Individual ROIs and a Model Gradient

**Figure 8:**
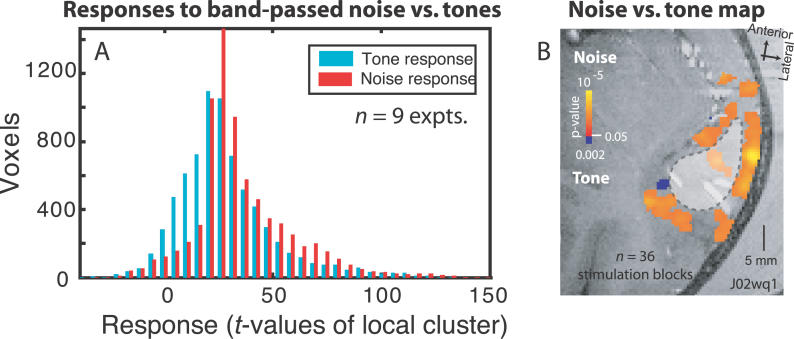
Comparing Noise to Tone Responses Reveals a Preference for Noise in the Belt In nine experiments we individually presented single frequency tones within the range of the comparison band-passed noise (see “Sound stimuli and presentation” in Materials and Methods) and compared responses. (A) Shows distributions of the auditory cortex response (see “MRI data analysis” in Materials and Methods) to tone or noise stimulation. The analysis is based on voxels significantly active to at least one of the stimulation conditions, i.e., under tone or noise stimulation. Voxels responded more to noise than tones (Wilcoxon rank sum test of the distributions, *p* < 0.0001). (B) Noise responses were larger than tone responses in regions of the auditory belt, as illustrated by this example experiment, confirming and extending predictions from neurophysiological recordings of the lateral belt [ 19, 21].

Furthermore, in two experiments with different animals, we localized ACFs using (one octave) band-passed noise (see Figure 7D–I). These results support that sounds with broader bandwidth can also be used to functionally tessellate auditory cortex and localize the many ACFs. The results with band-passed noise also confirm that tones activated large portions of auditory cortex, although band-passed noise generally gave stronger auditory cortex responses than tones (see Figure 8); compare the extent of the activity based on significantly active voxels in the multiple band-passed noise data from Figure 7G with the multi-tone data in Figure 5A, obtained during the same experiment.

The statistics from the ROI gradient tests for all of our multi-frequency experiments are summarized in Table 1. There we see that for the three ROIs representing the core fields A1, R, and RT, nearly all of the experiments revealed statistically significant gradients. The medial and lateral belt fields seemed more elusive because even neurophysiologically well studied fields like CM did not present a significant gradient in every experiment. Nonetheless, individual experiments supported the presence of four lateral belt fields (see middle of column 5 in Table 1) and four medial belt fields (see bottom of the last column in Table 1). Together, the data well support a model of 11 core and belt fields.

### Reliability of ACF Borders and Gradients: A Matter of Size

We first examined the within-experiment reliability of the experiment in Figure 5 by splitting the dataset in two ( Figure 5F– 5I). Similar gradient patterns as observed in the full dataset were also observed in the two split datasets (compare Figure 5G and 5I to Figure 5C). A correlation analysis demonstrated that the two multi-frequency maps shared a significant amount of variance ( *r* = 0.24, *p* < 0.001). This analysis supports that at least the large-scale patterns within auditory cortex are highly reliable. Looking more closely within individual ROIs of the split datasets, we noted that the gradients of the largest ROIs, that represent fields A1, CM, and CL, were significantly correlated across the split datasets (respectively, *r* = 0.41, 0.29, 0.17, all *p* < 0.05), whereas the other ROIs did not reach significance. To confirm that the ROIs were co-localized across the two datasets, we next computed their overlap. The mean overlap of ROIs defined for the split datasets was 74%, averaged across all ROIs, with a range of 89% overlap for the ROI representing field A1, and 53% for the ROI of field MM. These results show good overlap for the large fields in auditory cortex, but also reveal jitter in the borders attributed to the smaller fields.

Across-experiments with the same subject (see Figures 5C– 5E and 7A– 7C) we also observed good reliability of the data and overlap of ROIs. The gradients for the ROIs of the large fields A1, ML, and CL (see the volume measurements in Table 1) were significantly correlated across the two experiments (respectively, *r* = 0.32, 0.31, 0.23, all *p* < 0.01). Regarding ROI overlap, the ROIs defined independently for each experiment showed a mean overlap of 73%, and the range of ROI overlap across different fields was 80% overlap for the ROI representing field A1 and 43% overlap for the ROI of field RTM. These results demonstrate that the across experiment variability in the presumed tonotopic organization is comparable to that within an experiment.

Across all of the experiments, the volume attributed to the large fields showed a much smaller variance than for the smaller fields: The standard deviation of the ROI volume was 21% of the mean volume for A1, was only 7% for CL, but a high of 51% of the mean volume for RTL ( Table 1). Given that the ratio of border length to area is smaller for a large field compared to a small field, the same level of noise in establishing borders will affect smaller fields more than a larger field like A1. Therefore attempts to determine the borders of smaller fields, especially of belt fields whose frequency selectivity may be less consistent, will critically depend on the number of voxels within a field, and hence the resolution of fMR imaging.

### Hierarchical Processing in Auditory Cortex

Lastly, we used a different strategy to localize core fields in relation to belt fields, which unlike the method relying on only tone responses (as shown in Figure 5C), requires collecting further data. We contrasted the responses to tones versus band-passed noise as has been done neurophysiologically [ 19, 21, 27]. This strategy is based on the idea of a hierarchy of auditory cortex processing [ 1, 11, 19, 21, 27, 29], whereby the belt, being a hierarchically higher stage of auditory cortex than the core, is involved in the analysis of the features of complex sounds—belt neurons are thought to prefer sounds with a broader frequency spectrum, such as band-passed noise in comparison to single-frequency tones [ 19, 21, 27].

In nine experiments we compared the cortical responses to band-passed noise and single frequency tones, presented within the same frequency range as the comparison noise. Using tones to stimulate the same frequency range present in the noise aimed to rule out the possibility that broader fMRI responses to the noise can be trivially explained by a broader frequency spectrum of the noise activating a larger population of neurons than that responding to the tonal stimulation (see “Sound stimuli and presentation” in Materials and Methods). The results showed evidence for a distributed preference in the belt for band-passed noise in comparison to tones, consistent with the idea of hierarchical processing ( Figure 8). Also, the relative position of the core in relation to the belt can be approximated by the observed greater response to noise in the belt (see the outline in Figure 8B).

## Discussion

This study demonstrates reliable high-resolution functional imaging of the macaque monkey's auditory cortex. Non-invasive fMR imaging of monkeys can now be used to more directly compare the work obtained from imaging human audition and that obtained from neurophysiological and anatomical studies of the nonhuman primate auditory cortex. In combination with the recent positron emission tomography of macaque auditory cortex [ 31, 32], our work extends the range of techniques that can yield directly comparable data from human and nonhuman primate auditory cortex.

We next summarize and discuss our findings. Where appropriate we also consider how to extend these auditory fMRI results using neurophysiological and neuroanatomical techniques in the nonhuman primate.

### Functional Tessellation of Macaque Auditory Cortical Fields

These high-resolution fMRI experiments localized the activity from numerous small and proximally located macaque ACFs, providing an extensive functional tessellation of auditory cortex. For confirming a portion of the fMRI activity patterns, the results reliably revealed the six expected ACFs with available neurophysiological data (core: A1, R; belt: CM, CL, ML, and AL). We also delineated borders between these ACFs and other neighboring fields. Of these six auditory core and belt ACFs, the directions of the observed fMRI frequency gradients entirely support the neurophysiological evidence [ 16– 21]. Across experiments we commonly observed the frequency selective patterns and gradients that are expected for these six ACFs ( Table 1, also see Figures 5– 7).

Because of the broad activation of auditory cortex we obtained functional patterns from other auditory core and belt fields whose function is either poorly described or entirely unknown. For instance, because of difficulty electrophysiologically recording from the third auditory core field, RT, there is limited evidence of a tonotopic gradient in this field [ 2], which should mirror the one from its posterior neighbor, field R. Our results consistently revealed a significant tonotopic gradient in RT that mirrors the one in field R (see Table 1).

A number of belt fields have no available neurophysiological data and thus their anatomical parcellation has not been confirmed with functional data. For the lateral belt field RTL, our experiments often showed that the area lateral to field RT has a collinear tonotopic organization (see Figures 5– 7). This evidence is perhaps not surprising, given that the other lateral belt ACFs (fields CL, ML, and AL) have collinear tonotopy in relation to their medial neighbors (CM, A1, and R, respectively, e.g., see Figure 5). In one experiment the gradients of all four of these lateral belt fields were significant (see middle of column 5 in Table 1), providing a functional confirmation of these four anatomically described lateral belt fields [ 1, 3], of which only the posterior three have been neurophysiologically described [ 19, 21, 29]. The gradient maps of other experiments were supportive (e.g., see middle of column 4 in Table 1) although certain experiments could only significantly implicate some of these fields' gradients ( Table 1).

Regarding the medial belt fields, it is possible that many neurons here are also frequency selective like their lateral counterparts. This would suggest that prominent properties like frequency selectivity guide the overall organization of the core and belt regions in auditory cortex. Partial support for this is already available because the belt field CM has a neurophysiologically well characterized tonotopic gradient that is collinear relative to its lateral belt neighbor, field CL (see Figure 2D and [ 16, 19– 21]). As already discussed, our fMRI data support this (see Figures 5– 7 and Table 1). Notably, where the medial iso-frequency bands and gradients appeared clearer, the fMRI data show that the iso-frequency bands first observed in A1, R and RT also extended medially (see Figures 5 and 7G, 7H). Such results are consistent with some of the interpretations of the anatomical evidence that, rather than just three medial belt fields (i.e., CM, RM, and RTM), there could be a total of four medial belt fields: CM, MM, RM, and RTM [ 1, 3]. Such an organization of the medial belt is appealing because it complements the four lateral belt fields. In one experiment we confirmed that the gradients of all four of these medial belt fields were significant (see lower portion of the last column in Table 1). Inspection of the gradient maps from the other experiments were supportive (see lower portion of column 4 in Table 1), although not every experiment could significantly implicate the gradient of each of these medial fields.

Many of our results were well replicable and our detailed analyses of the 11 ROIs showed good overlap in ACF locations within and across experiments with the same subject. The analyses also suggested that the statistics of the small fields' gradients, the belt fields in particular, did not always turn out significant because variance in border estimation seemed to favor the larger fields with more voxels. Thus it seems unrealistic to expect that every experiment will statistically identify so many small and proximally located regions of the brain, although some individual experiments certainly came very close to achieving this goal. It is also possible that ACF borders are not as strict as we assume them to be, in which case a combination of techniques besides functional imaging would be needed to establish “borders” (see “Delineating Borders across Auditory Cortex with fMRI” in Discussion).

Our experiments initially relied on the known ACF anatomy and neurophysiology in these species to confirm portions of the obtained fMRI activation patterns. Upon this basis, activity patterns from other portions of auditory cortex were also identified. The findings extend our understanding of auditory cortical processing, especially from the less functionally known fields in the core, lateral and medial belt regions of auditory cortex. Specifically, our results support a model of 11 tonotopically organized core and belt fields.

### Tonotopy in Auditory Cortex with fMRI: Considering Sound Intensity

Tonotopy is considered a basic functional property of many primate ACFs [ 16– 20] and was prominent with fMRI. This was the case even at moderate to high sound intensities where many neurons become broadly tuned and tonotopy deteriorates neurophysiologically [ 30]. For this reason neurophysiological maps of best frequency are usually obtained close to a neuron's threshold or at low sound intensities. Others have also noted this for human auditory fMRI where setting sound intensities to a moderate 70 dB SPL revealed tonotopy over the human homologues of A1 and R [ 13]. Our work by design used moderate to high sound intensities (70–85 dB SPL) in order to significantly activate large portions of auditory cortex. Thus even tone activations were broad considering that belt neurons are not very strongly driven by tones [ 16, 19– 21]. Nonetheless, upon such a large active area, frequency selectivity was observed, supporting the observation of multiple tonotopic maps in humans obtained at moderate sound intensity levels [ 13].

### Hierarchical Processing of Auditory Cortex

We also observed a distributed preference for band-passed noise over tones in the auditory belt, the second stage of auditory processing in the neocortex. This result is consistent with the idea of hierarchical processing which posits that belt neurons prefer complex sounds, such as those containing a broader frequency spectrum than single-frequency tones [ 1, 11, 21, 27, 29, 33]. Our results with monkeys complement the prior fMRI study with humans [ 11] and the combined fMRI results from these primates provide a strong demonstration of similar principles of hierarchical processing in the primate brain. Furthermore, the two studies suggest that both lateral and medial belt regions of primates prefer band-passed noise over single frequency tones, complementing our other finding that lateral and medial belt fields are tonotopically organized. Thus some similarities in organizational principles of the auditory cortex seem to exit. However, it is also expected that, within a framework of related organizing principles, many ACFs will contribute differently to the analysis of the acoustical environment. For instance ACFs are expected to have specialized roles, such as the selective processing of monkey communication sounds or their spatial location (e.g., see [ 33, 35] for ideas of such specialized processing from neurophysiological recordings of the macaque lateral belt).

### Delineating Borders across Auditory Cortex with fMRI

We functionally demarcated a border around core and belt regions, which neurophysiologically has only been studied in part because of the large extent of this border. Traditionally, anatomical/histological techniques have been used to delineate, with good certainty, this border around primary auditory cortex. This is done in part by identifying the granular (koniocortical) cytoarchitecture, including denser staining of myelin and other elements present in greater density in the primary sensory cortex [ 3, 17, 18, 22]. We showed that different fMRI approaches can be used to approximate the border between the core and belt regions. These included using strategies motivated from neurophysiology, such as a thresholded tone response or by identifying stronger noise responses in the belt. Now, direct comparison of fMRI and anatomical data from the same animal will show whether the two techniques exhibit close correspondence in their description of the border between primary and non-primary auditory cortex (i.e., core and belt regions).

Regarding the borders delineated using tonotopic gradients, our data reveal a fair amount of within subject variance in the position of ACF borders based on tonotopy. Using similar fMRI methods, others also show some variance in the border between A1 and R with the same human subject [ 13]. Since these relatively short stretches of borders between individual ACFs are also commonly described with neurophysiological and anatomical techniques, a reasonable first question is which approach or approaches best estimate these borders, assuming that strict borders exist between fields. Addressing this question in the same nonhuman primate by comparing ACF borders described by fMRI, neurophysiology and neuroanatomy can help us to understand whether we can define ACF borders by a technique independent standard, or whether strict borders are primarily a theoretical concept.

### Considerations for Auditory Studies Using fMRI and Neurophysiology

The capability to record from single neurons in monkeys in combination with high-resolution fMRI can now broaden our understanding of auditory cortical function. The combined approach makes a powerful combination to address questions of function because, as shown here, fMRI can reveal properties that would be difficult to detail neurophysiologically. Alternatively, recording from single neurons is the standard for understanding function at the single unit level that could easily be overlooked by pooling across a population of active neurons.

Functional auditory data from one technique can now guide the design of experiments for use with the other. For instance, carefully designed neurophysiological experiments can now be used to elicit robust responses from neurons in auditory cortical fields where neurophysiological recordings have not been successful. Such experiments will support or clarify our observations from these fields. Alternatively, current knowledge from neurophysiology can guide further fMRI experiments. In this case, ACF differences in sound processing, across certain core and belt fields in primates [ 29, 34] or across the antero-posterior extent of the lateral belt fields [ 33, 35], can now be evaluated with fMRI for many fields in the auditory cortex. Furthermore, we didn't use band-passed noise bandwidths smaller than one octave in spectral width, unlike electrophysiological work [ 19, 21]. Imaging experiments can now study the bandwidth preference of auditory cortex to determine if there is a preference for larger stimulus bandwidths in the hierarchically higher auditory regions, analogous to an expansion of visual receptive fields in higher visual areas.

However, care should be taken in interpreting data from fMRI or when considering how to adapt fMRI experiments for neurophysiology [ 13, 36, 37]. For instance, our multi-frequency maps reflect the strongest BOLD response to a specific frequency. Yet, at these moderate to high sound intensities, other frequencies also activated large portions of auditory cortex. Thus two frequency maps based on tones spaced at least four octaves apart (see the two-tone maps in Figures 3 and 4) often showed the clearest differences in frequency selectivity. These observations suggest that neurons were broadly tuned to these stimuli, especially at the intensity levels that are necessary to obtain robust fMRI activity throughout auditory cortex.

### The Efficiency of Localizing ACFs

Rapid localization is important as a basis for clarifying further auditory imaging. Therefore, how quickly can ACFs be localized? The data presented in Figure 5 were obtained after averaging many repeats of the same 25-min stimulation sequence. There we functionally tessellated auditory cortex and delineated borders between ACFs using six tone frequencies, including using the tone activity to help to localize the core versus belt regions. However, we observed similar results with a split data set suggesting that the process could have been shorter. Surprisingly, analyzing individual 25-min runs (with only three repeats of each stimulus) showed very similar results, providing that the activity was strong enough (see Figure S1 online). These observations are promising for further expediting the localization of the many ACFs (see [ 38] for expedient procedures for visual field localization).

### Advancing the Search for the Human Auditory Cortical Fields

The close proximity and the small scale of the many auditory fields have undoubtedly made it difficult to localize them in humans, in contrast to visual studies that currently benefit from the ability to localize numerous visual fields in both humans and macaques [ 6, 7]. The scale of sensory fields is even more problematic for the smaller macaque brain where it is critical to attain sub-millimeter voxel resolution for imaging auditory fields that can be smaller than 5-mm in length or width. Thus for localizing multiple ACFs, high-resolution imaging at high-magnetic fields [ 39, 40] seemed instrumental in our experiments and also for the human study [ 13]. Additional factors contributing to the obtained results involved optimized imaging and sound stimulation procedures. The combined contribution of this work should facilitate further auditory functional imaging at high magnetic fields.

These macaque results now offer the possibility of using strategies similar to ours to localize further human ACFs with fMRI [ 13]. A comparative approach in these primates with identical imaging and stimulation protocols could be invaluable regarding the location, orientation and number of evolutionarily conserved ACFs [ 7, 41]. The homology of fields is the minimum knowledge required for understanding how the specialized areas—for communication sounds or the language areas in humans—evolved and what their dependence is on the initial processing stages of auditory cortex. Although, high-resolution fMRI is in principle not necessary for addressing the homology problem over larger portions of auditory cortex [ 31], given the scale of many primate ACFs, high-resolution imaging seems important for addressing the homology of specific fields or portions of auditory cortex.

In summary, high-resolution fMRI revealed spatially resolvable activity from the auditory cortex of the macaque monkey. This allowed a functional tessellation of many auditory cortical fields, including those whose functional properties were previously unknown. Localized patterns of auditory cortex activity can now clarify the source of subsequent imaging of this part of the brain. Auditory fMRI will compliment other techniques commonly used in the nonhuman primate to extend our understanding of the primate auditory cortex.

## Materials and Methods

This study obtained data from 26 fMRI scanning sessions with seven male macaque monkeys *(Macaca mulatta),* weighing 5–8 kg, six of which were scanned multiple times. Six macaques were scanned under anesthesia and one while performing a visual fixation task, assisting the animal in maintaining minimal body movement during scanning. All studies were in full compliance with the guidelines of the European Community (EUVD 86/609/EEC) for the care and use of laboratory animals and were approved by the local authorities (Regierungspräsidium). All studies were done with great care to ensure the well-being of the animals.

Measurements were made on vertical 4.7- and 7-T scanners (Bruker Medical, Ettlingen, Germany), see below, “MRI hardware and imaging.” Results were comparable across scanners when the same animal was scanned using identical procedures (compare Figure 5 to Figure 7A–C).

### Animal preparation.

A primate chair was used for positioning the animal within the magnet. During the experiment, the animal's head was positioned with a custom-made plastic head holder (Tecapeek material, Ensinger GmbH, Nufringen, Germany) onto small attachment points previously implanted on the cranium.

### Behaving animal preparation and imaging.

For behaving-animal imaging at 7-T we trained a macaque (J03) to complete trials of visual fixation in combination with minimal body movement. This involved over 6 mo of shaping the animal's behavior using operant conditioning; the animal was on a reduced water access protocol and juice served as the behavioral reward. In a mock environment, the animal was trained to tolerate the gradient noise and the limited space of the cylindrical chair. During scanning, a trial began following minimal motion for 4 s on jaw and body sensors. Then the animal engaged a visual fixation dot that was presented with an SVGA fiber-optic system (AVOTEC Silent Vision, Stuart, Florida, United States). Eye movements were measured with a custom made infra-red eye tracker. Following fixation, a sparse-imaging sequence began, similar to the one used for anesthetized animal scanning ( Figure 1B). A volume was acquired, and then 3 s after the initiation of fixation sound stimulation began, which lasted for 5 s. At 10 s following fixation the stimulus-related volume was acquired after which the animal was rewarded for correctly completing the trial. Eye or body movements aborted the trial. Only correctly completed trials were analyzed.

### Anesthetized animal preparation.

An extensive description of the handling and anesthesia procedures can be found elsewhere [ 42– 44]. The handling and anesthesia protocols ensure stress-free treatment of the animal, while, at the same time, preserve neural responses to sensory stimulation. After premedication with glyco-pyrolate (i.m. 0.01mg per kg) and ketamine (i.m. 15 mg per kg) a catheter was inserted into the saphenous vein. Animals were preoxygenated and prepared for intubation with a cocktail of short-acting drugs (fentanyl at 3 μg per kg, thiopental at 5 mg per kg, and the muscle relaxant succinyl-choline chloride at 3 mg per kg). The trachea was then intubated and the lungs ventilated at 25 strokes per min. Anesthesia was maintained with remifentanyl (0.5–2 μg per kg per min) and muscle relaxation with mivacurium chloride (5 mg per kg per h). Lactated Ringer's solution was given intravenously at a maximum rate of 10 ml/kg/h. Physiological parameters (heart rate, blood pressure, blood oxygenation, and expiratory CO _2_) were monitored and kept in desired ranges with volume supplements. Body temperature was regulated using circulated water heaters. Functional data acquisition started approximately 2 h after the start of the animal preparation following a high resolution anatomical scan.

### Sound stimuli and presentation.

Sound stimuli were single-frequency tones or noise bursts, ranging in spectral composition from 1-, 2-, and 3-octave band-passed noise to broadband noise (0.250–19 kHz). The tone and band-passed noise stimuli allowed us to vary the center frequency of sounds within each stimulus group. For experiments where sounds from a single group were used, the exact frequency composition of the sounds used is identified in the reported experiments. In some experiments we also varied the spectral density by using sounds across groups. For such experiments where we compared responses to single frequency tones and band-passed noise ( Figure 8), we randomly varied the frequency of a single frequency tone throughout the stimulation block so that the combined frequencies of the tones were within the spectral range of the comparison noise, using a frequency spacing of half an octave.

All sounds were 50 ms in duration, were sampled at 44.1 kHz and presented at 8 Hz (75-ms inter-stimulus-interval). Their amplitude was cosine-shaped on and off for 8 ms. Sound intensity levels were in the range of 70 and 85 dB SPL. For experiments with anesthetized animals, short scanning runs were first probed with broadband noise at intensity levels varying between 70–85 dB. This allowed us to select a minimum level within this range to obtain broad auditory cortex activity (˜3 cm, antero-posteriorly). With subsequent stimulation at this preselected intensity value, we balanced between obtaining broad activation of auditory cortex and preserving neuronal frequency tuning. Exceeding 85 dB SPL during sparse imaging was unnecessary because frequency selectivity was not observed at higher sound levels. For behaving-animal imaging the intensity level was set at 80 dB. For the combined tone and noise experiments the stimuli were RMS matched and presented at the same intensity level, in dB SPL.

Sounds were presented using custom written software and controlled using a QNX real-time operating system (QNX Software Systems, Ottawa, Canada). Sound stimuli were stored as WAV files, played from a PC, amplified (Yamaha, AX-496, Yamaha Worldwide, Hamburg, Germany) and delivered using MR-compatible headphones (MR Confon GmbH, Magdeburg, Germany). The headphones are composed of an electrodynamic membrane [ 45] within customized and foam insulated headphone cups, customized for use with these monkeys. The headphones were secured over the monkeys' ears and covered with foam (Tempur-Pedic, Tempur Deutschland GmbH, Steinhagen, Germany) to further attenuate outside sounds. Calibrations and sound intensity measurements were made with an MR-compatible condenser microphone (Brüel & Kjær 4188, Brüel & Kjær GmbH, Bermen, Germany) and a sound level meter (Brüel & Kjær 2238 Mediator, unfiltered calibration). Each headphone was individually calibrated to ensure a linear transfer function (from 0.088–19 kHz). The scanner noise was measured at a maximum of 105 dB SPL, see next section, which is estimated to peak at around 80 dB SPL at the ear, following approximately 25 dB of attenuation by the headphone cups and surrounding foam.

### MRI hardware and imaging.

The 4.7-T scanner is equipped with a 40-cm diameter bore (Biospec 47/40v, Bruker Medical, Ettlingen, Germany) and a 50 mT/m actively shielded gradient coil (Bruker, B-GA 26) of 26 cm inner diameter. For the scanning sequences used here, this scanner's noise peaked at 105-dB SPL. The 7-T scanner is equipped with a 60 cm diameter bore (Biospec 7/60v, Bruker) and an actively shielded gradient coil (B-GA 38, Bruker) of 38-cm inner diameter. Because of the larger bore diameter, the behaving animal was imaged exclusively in the 7-T scanner. The gradient for the 7-T scanner can achieve a maximum of 80 mT/m gradient strength per channel in less than 200 μs. To reduce the noise from this gradient, the scanner has acoustical shielding (33-cm effective bore diameter) that reduces the scanner noise approximately 30 dB to a measured maximum of 100 dB SPL.

Signals were acquired using a 70- or 80-mm diameter surface radiofrequency coil positioned over the auditory cortex of one hemisphere. To obtain a plane of activity corresponding to auditory cortex, slices were oriented parallel to the lateral sulcus (see Figure 2A) by relying on sagittal anatomical images obtained with a FLASH sequence. With this imaging sequence we used the following parameters, for anaesthetized animals: (TE: 6 ms, imaging sequence repetition time [TR]: 500 ms, spectral width of 25 kHz, field of view (FOV): 9.6 × 9.6 cm ^2^, on a grid of 128 × 128 voxels, 2-mm slice thickness, 15 slices), or for the behaving animal: (TE: 15 ms, TR: 750 ms, the other parameters were the same). This sagittal anatomy was also used to align a volume, centered on auditory cortex, for optimizing the linear and higher-order shim coils using an autoshim algorithm [ 46].

Functional data were acquired using multi-segment (multi-shot) gradient-recalled echo-planar imaging sequences (GE-EPI). For anesthetized animals the following parameters were used for sparse-imaging: four segments, TE: 16 ms, volume acquisition time (TA): 1.5 s, TR: 10 s, spectral width 100 kHz, 128 × 128 voxels, 2-mm slices, six slices. Behaving animal GE-EPI parameters were: two segments, TE: 19 ms, volume TA: 1.5 s, TR: 10 s, 96 × 96 voxels, FOV: 9.6 × 9.6 cm ^2^, 2-mm slices, four slices. For the anesthetized animal experiments the FOV was adjusted for each animal and was between 6.4 × 6.4 cm ^2^ and 12.8 × 12.8 cm ^2^. This resulted in voxel sizes of 1–2 mm ^3^. Thereby, to allow a better comparison between experiments, activations are expressed in volume units of ml and not as absolute numbers of voxels ( Table 1).

Anatomical images (T1-weighted) used the same planar FOV as the functional scans allowing anatomical slices to be in register with the functional slices that were acquired during the same imaging run. Anatomical volumes were acquired for the anesthetized animals with an eight-segment 3D-MDEFT sequence (three-dimensional modified driven equilibrium with Fourier transform pulse sequence, TE: 4 ms, TR: 22 ms, 256 × 256 × 128 voxels, with a typical FOV of 9.6 × 9.6 × 6.4 cm ^3^). For anatomical imaging of the behaving animal we used a FLASH sequence with the following parameters (TE: 15 ms, TR: 750 ms, 192 × 192 voxels, 2-mm slices, four slices). Displayed images are in radiological coordinates where left is right and right is left.

### MRI data analysis.

The data were analyzed off-line using custom written programs in Matlab (Mathworks, Natick, Massachusetts, United States). Multi-slice data (volumes) were converted to time points and linear drifts removed. Responses to sounds were quantified from repeated runs of stimulation blocks with flanking no-stimulation “baseline” blocks, or of correctly completed trials for behaving-animal imaging, where the first acquired volume served as the no-stimulation baseline. Functional maps or stimulus-related responses were computed using cross-correlation with a boxcar-shaped, zero-phase shift waveform, convolved with a gamma function [ 42].

To determine active voxels for further analysis we used a permutation test taking into account both voxel response and spatial cluster extent [ 47– 49]. Here we used the metric “cluster mass” [ 47– 49] which was calculated for each voxel as the sum of its and immediate neighbors' activation values (named *t*-values of local cluster in Figure 8A). A permutation test (boot-strap procedure) then determined the significance level ( *p*-value) based on a randomized time series subjected to the same clustering procedure. The advantage of the permutation test is that it naturally combines information about neighboring voxels, thus incorporating cluster size into the significance test and reducing the need for post-hoc clustering or *p*-value correction. Activations were considered significant below *p* = 0.05, see Figure 2B. This analysis and the local signal obtained from the surface coil resulted in significant activations that were highly localized over the grey matter of the superior temporal plane. Therefore, activity was over a limited number of slices (1–3, 2-mm slices) and results could be averaged over these slices, or, in cases of optimal slice positioning, presented from a single slice. Only significantly active voxels to the specific sounds under analysis were further analyzed.

Frequency selectivity was determined by taking the difference of the stimulus correlation between two frequency conditions. Multi-frequency maps show for individual voxels the frequency eliciting maximal response. Similar results were obtained from the maximal response of Gaussian fits to voxels' multi-frequency responses. For use in the gradient-sign analysis the multi-frequency data was first smoothed using a 2-dimensional Gaussian filter (3 voxel half-width at half maximum). An ROI around the significantly active voxels limited the smoothing within this region of auditory cortex. The gradient-sign mapping procedure (also see the text), assigns to voxels a positive or a negative gradient value (in the selected antero-posterior axis) based on the frequency response of the immediate neighboring voxels. Small deviations in the axis direction (< 20°) or just using the animal's own antero-posterior axis showed similar gradient patterns and closely corresponding borders. Orthogonal axes in the medio-lateral direction, however, resulted in exceptionally noisy patterns.

The ROIs that we outlined were based on the results from the gradient sign maps. However, we also strove to stay within a reasonable structure of the 11 proposed ACFs, see [ 1] and right panel in Figure 5A.

## Supporting Information

Figure S1 Individual Scanning Runs with Six Individually Presented Tone Frequencies Reveal the Expected Spatially Alternating Pattern of Frequency Selective Regions (e.g., see Figures 5– 7)
(A–C) The analyses are from the experiment shown in manuscript Figure 5. Each is based on a 25-min run where data from three stimulation blocks per tone frequency were obtained, including flanking baseline blocks. The three scanning runs shown were selected for having the most activity of all individual scanning runs. Here the results include some subthreshold activity because we display the best-tone frequency response of all of the voxels identified as active in the entire experiment (see Figure 5). The observed patterns of frequency selectivity shown here were not evident in the remaining scanning runs with less activity, suggesting that much of the subthreshold activity was noisy.
(2.3 MB EPS)

Protocol S1 Reliable Patterns of Frequency Selectivity (29 KB DOC)

## Abbreviations

ACF: auditory cortical fields
BOLD: blood-oxygen level dependent
fMRI: functional magnetic resonance imaging
ROI: region of interest
SPL: sound pressure level
T: Tesla
