# Supplementary material for: Functional Imaging Reveals Numerous Fields in the Monkey Auditory Cortex
Source: PLoS Biol. 2006 Jun 20;4(7):e215. doi: 10.1371/journal.pbio.0040215 (PMC1479693; doi:10.1371/journal.pbio.0040215)
Supplement: Supplementary file 1 [file pbio.0040215.sd001.doc]

**Supplementary Online Materials**

“Functional Imaging Reveals Numerous Fields in the Monkey Auditory Cortex”

Christopher I. Petkov, Christoph Kayser, Mark Augath & Nikos K. Logothetis

**Protocol S1**

**Supporting notes - Reliable patterns of frequency selectivity**

The antero-posterior alternating pattern of four low and high frequency selective regions (see manuscript Figs. 3 and 4) was reliably observed: (1) across experiments, (2) by using different sounds, e.g., tones or noises, and (3) with sounds of different spectral composition. Nine out of 14 tone experiments showed the two pairs of low and high frequency selective regions (see manuscript Figs. 3 and 4 for examples). Eight out of 13 experiments with band-passed noise, composed of bands of 1-, 2- or 3-octaves, also revealed this pattern of frequency selective regions. Many multi-frequency experiments, with tones or noise, revealed an extended pattern that included a more posterior low-frequency selective region from the posterior belt, e.g., the region posterior to label H1 in manuscript Figure 4C, also see manuscript text and Figs. 5-7.

For the remainder of tone or noise experiments, due to a lack of strong activations, less than the two pairs of low and high frequency selective regions were revealed. With such limited activity and number of frequency selective regions it was difficult to unambiguously associate the obtained activity patterns with specific ACFs, see manuscript text.
